# Measurement and control of magnetic thin films and devices using thermal gradients applied via suspended Si-N membranes

**DOI:** 10.1080/14686996.2025.2531735

**Published:** 2025-07-23

**Authors:** B. L. Zink

**Affiliations:** Department of Physics and Astronomy, University of Denver, Denver, CO, USA

**Keywords:** Seebeck Coefficient; thermal conductivity, thin films, spin transport

## Abstract

Magnetic thin films and nanostructures present a unique challenge for a range of thermal measurements, with important consequences for both fundamental physics and material science and applications. This paper reviews the unique capabilities for measurement and control of these systems using thermal gradients applied using micro- and nanofabricated silicon-nitride membrane platforms. Supporting a thin film or nanostructure removes bulk heat sinks from the tiny structure, enabling otherwise challenging or impossible measurements including thermal conductivity, Seebeck coefficient, Peltier coefficient, magnon drag, both the anomalous and planar Nernst effect, specific heat, and novel manifestations of thermally assisted spin transport. After providing some historical context and motivation and overviewing the design and fabrication of silicon-nitride membrane thermal platforms, example data for each of the measurements above is reviewed, and the paper concludes with a consideration of the outlook for measurements enabled by these techniques.

## IMPACT STATEMENT

This review summarizes measurements of magnetic films enabled by Si-N membrane techniques.

These probe properties including thermal conductivity, magnon drag, anomalous Nernst effects, and thermally driven or assisted spin transport.

## GRAPHICAL ABSTRACT

**Figure:**
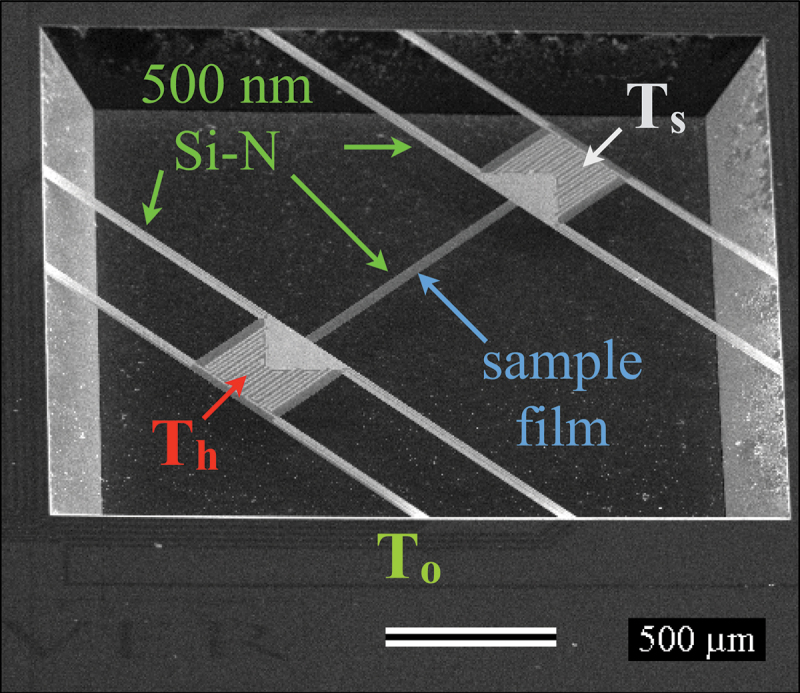

## 1. Introduction

The fundamental science concerning the interaction of heat and magnetism, while not as old as humanity’s knowledge of ferromagnets, dates to our earliest meaningful attempts to experimentally quantify and understand magnetism. In perhaps one of the earliest and most impactful scientific works on magnetism published in 1600, Gilbert overviewed experimental knowledge of the loss of magnetism in a ferromagnet upon heating, now of course known as the phase transition from ferromagnetism (FM) to paramagnetism at the Curie temperature [1]. Much later experiments during the late 1800s by Nernst used thermal gradients applied to bulk metals to demonstrate the link between magnetic and electric fields, leading to our current understanding of the ordinary and anomalous Nernst effects [2–4].

Applying thermal gradients remains an important approach both for studying the fundamental properties of a broad array of materials with various magnetic order [5–8] and for potential magnetic and spintronic applications. Such applications range from energy harvesting [9–14] to information technology [15–20]. Many materials and systems of current and future interest exist only in the form of thin films and nanostructures. These tiny structures present both challenges and opportunities for understanding their novel fundamental properties and using them for new device applications.

**Figure 1.**
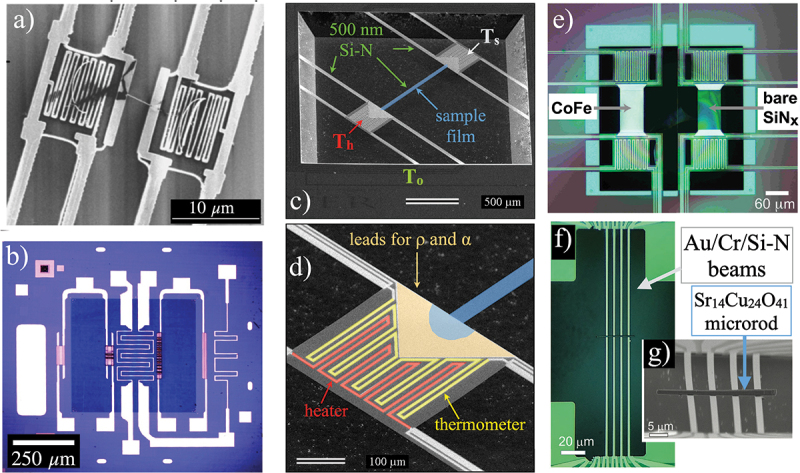
(a) A two-island Si-N structure used to measure heat transport in individual carbon nanotubes (image modified from [21] with permission of the authors, copyright 2001 by the American Physical Society). (b) A Si-N nanocalorimeter with Pt heaters and thermometers, and Nb-Si low temperature thermometers designed for thin film heat capacity measurements [22–26]. (c) Scanning electron micrograph of a suspended Si-N platform for thin film k, ρ, and α measurements [27–29]. Here two suspended islands are bridged by a narrow sample platform (shown with blue highlight). (d) A closer view of one island of the platform shown in (c), with false color highlights indicating the lithographically patterned heater (red), thermometer (yellow), and sample leads (orange). (e) Optical micrograph of a matched pair of suspended Si-N thermal platforms, one that serves a reference while the other supports a metal sample, here a Co-Fe alloy film (image modified from [30] with permission of the authors, copyright 2018 by the Institute of Physics). (f),(g) A more recent design of a suspended Si-N platform optimized for nanoscale samples, here with microrods of the spin-ladder compound Sr${\;}_{14}$Cu${\;}_{24}$O${\;}_{41}$, used to demonstrate a magnon contribution to k (images modified from [31] with permission of the authors, copyright 2020 by Wiley).

This paper reviews the control and understanding of magnetic systems possible using applied thermal gradients, with a special emphasis on materials in the form of thin films or nanostructures. The central focus is on the use of micro- or nanomachined platforms where a tiny magnetic system or device is suspended on a silicon-nitride (Si-N) membrane. Silicon-nitride (sometimes abbreviated Si-N${\;}_{x}$ to explicitly point out that films are often made with differing stoichiometry from the bulk ceramic material Si${\;}_{3}$N${\;}_{4}$) is a strong and highly tunable material that can be grown in thin-film form in several ways. Growth of suspended membranes, such as shown for a range of thermal measurement devices in Figure 1, almost always employs material grown via low-pressure chemical vapor deposition (LPCVD) at elevated temperatures, since adjusting the ratio of the precursor gases allows simple tuning of the ratio of Si and N atoms in the resulting film, which in turn adjusts the residual stress in the film [32,33]. This stress is tunable over a very wide range, from compressive to tensile, which allows near-zero stress to be achieved. In such films, if the bulk substrate supporting the Si-N (often Si) is removed from selective areas, the Si-N forms a stable and strong membrane supported by the remaining Si frame. Such structures have important applications in micromechanical systems (MEMS) and actuators [34].

Such Si-N membranes were first applied to fundamental materials physics in the form of micromachined calorimeters for measurements of the specific heat capacity and heats of transformation [22–26,35–41]. Parallel work focused on similar structures for low-temperature photon detectors and bolometers for astronomy, cosmology, and other sensing applications [42–46]. The ability to fabricate patterned suspended Si-N structures with integrated thermometers and electrical leads enabled dramatic measurements of the thermal and thermoelectric properties of nanostructures such as carbon nanotubes starting in the early years of the 21${\;}^{\mathrm{st}}$ century [21,47–50]. This approach helped open new areas of thermal transport in nanostructures. Much of the focus of this review is on thermal isolation platforms optimized for deposition of thin-film materials on a narrow sample platform bridging a two-island structure, inspired by devices such as the one shown in Figure 1(a). The main motivation for this approach is to measure in-plane thermal conductivity in a wide range of thin-film materials that can be deposited on the suspended Si-N. A very important additional advantage is the addition of electrical leads that also contact the sample (both in the traditional longitudinal geometry used for resistivity measurements and the transverse geometry that enables Hall and Nernst measurements). This allows for unambiguous examination of the Wiedemann-Franz law for a single thin-film sample.

Measuring the in-plane electrical conductivity of a thin film or nanostructure supported by a bulk substrate is commonplace and straightforward, because common electrically insulating substrates or thin-film coatings (such as SiO${\;}_{2}$ or Si-N) exist with electrical conductivity many, many orders of magnitude lower than the sample. For example, even in the thin-film form, SiO${\;}_{2}$ can have electrical conductivity $\sigma ∼10^{-16}\;(\Omega \;\mathrm{cm}{)}^{-1}$ [51], making charge leakage into this material utterly negligible for most experiments. The situation for thermal conductivity, k, is very different, since k ranges over only about 3 orders of magnitude for typically available materials [52]. This means that accurate measurements of the thermal conductivity of a thin film supported by a bulk substrate are almost always impossible in practice. For example, bulk-disordered SiO${\;}_{2}$ (glass) has k∼1W/mK near room temperature [53,54]. As the experiments reviewed below show (Figure 5(a)), typical transition metal ferromagnetic thin films have k∼25W/mK near room temperature. Keeping in mind that typical substrates are 10,000× thicker than films of interest for both fundamental studies and applications, this suggests the parallel thermal conductance of the substrate, $K_{\mathrm{sub}}$ (we define thermal conductance in analogy to the electrical conductance case, K=kA/L, where A is the cross-sectional area of the heat conducting material and L is the length of the heat conductor [56]) in an experiment where a 75nm thick FM film is deposited on a 0.5mm thick substrate, $K_{\mathrm{sub}}=267\times K_{\mathrm{film}}$. The substrate therefore completely thermally shorts the film. Measurements of k for thin films therefore either must use techniques which naturally avoid heating the substrates, such as the powerful and popular optical pump-probe-based measurements such as time-domain thermoreflectance [57,58], or where the substrate is reduced or eliminated [59–68]. The techniques I overview here using Si-N membranes for magnetic systems fall into the latter category.

The techniques required to reliably measure k for thin films using Si-N membranes have found natural extensions for additional measurements and control of magnetic films using the thermal gradients generated in the suspended structure. Many of these are enabled by the largely unambiguous direction of thermal gradients applied to a suspended Si-N structure, which is overwhelmingly in-plane since the bulk heat sinks provided by the substrate and some methods for contacting thin films on substrates have been removed. These issues are discussed in more detail elsewhere [69]. After providing a short discussion of the nanofabrication techniques used to produce Si-N membrane thermal isolation platforms, this review first overviews thermal conductivity measurements, focusing mostly on magnetic films. We then move on to a discussion of several of these other measurements or means of thermal control of magnetic systems and devices that are enabled by the Si-N membrane approach, before closing with some indications of important future directions for these techniques.

## 2. Design and fabrication

There are two fairly common methods of bulk Si micromachining that have been applied to the production of the patterned Si-N structures used in the work reviewed here, one using an anisotropic wet etch of Si, typically either using heated potassium hydroxide (KOH) or tetramethylammonium hydroxide (TMAH), and the second using the Bosch process deep trench dry etching of Si. In some work, patterned Si-N structures were formed from commercially available Si-N membranes, with the metal films that form heaters, thermometers, and samples put in place before a plasma etch step is used to pattern the Si-N membrane [30]. The choice between these approaches is typically driven by compatibility of various elements of the platforms with the aggressive KOH or TMAH etch needed to form the membrane if the wet-etch process is used.

**Figure 2.**
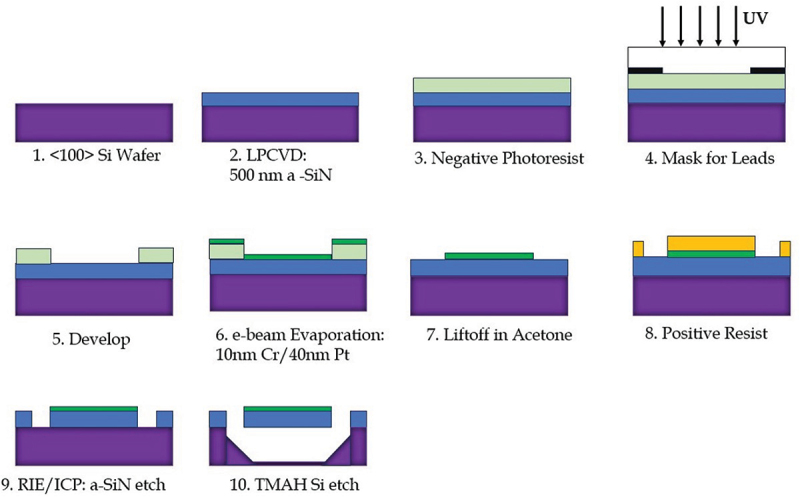
Schematic overview of steps to produce suspended Si-N membranes by anisotropic Si wet-etching. (1) processing begins with a <100> oriented Si wafer. (2) Si-N is grown (on both sides, note only the front side is visible in the schematic) via LPCVD. (3) Negative photoresist is spun on the Si-N/Si top surface and (4) patterned via optical lithography to form the windows that define the lead, contact and thermometer pattern. After development, (5), the Cr/Pt metal layer is deposited via e-beam evaporation, the metal layer forming the heaters and thermometers will be patterned via negative photoresist.

A typical wet-etch process flow, which is overviewed schematically in Figure 2, begins by depositing low-stress Si-N on a (100) oriented Si wafer, which can be single-side polished, via LPCVD as described above. The most common process for this uses ammonia and dichlorosilane precursor gases, and a deposition temperature near $800^{\circ }$ C. After Si-N growth, with the thickness setting the eventual thickness of the suspended membranes, metal patterns to form the heaters, leads, and thermometers are patterned via photolithography on the front of the wafer. Here, the entire stack must be compatible with the later KOH or TMAH etch. Films that have been demonstrated with this technique include sputtered molybdenum films with thickness 200 nm, and sputtered ∼40 nm platinum films with a sputtered ∼10 nm thick-chromium sticking layer. After patterning of the metal (either via chemical etching or by photoresist liftoff), the Si-N is selectively removed from the front of the wafer in a pattern that defines the shape of the membranes. After windows are etched through the Si-N using SF${\;}_{6}$ or similar plasma reactive ion etching to expose the bare Si below, the wafer is etched in heated KOH or TMAH (typically at $80-90^{\circ }$ C, though this can be adjusted to achieve a particular etch rate). These etches attack any plane of crystalline Si other than the (111) plane rapidly. The Si-N also has high resistance to this etch and acts as a very effective mask to protect Si. It is usually preferable to allow the etch to proceed long enough to fully detach the thermal isolation structure from the wafer but not long enough to etch the entire wafer for subsequent ease of handling. Rinse of the Si etchant in water must proceed with some care, as the suspended Si-N structures are fully formed after the etch, and in some cases, it can be beneficial to add a final immersion in a weak solvent such as ethyl or methyl achohol before drying the membranes, since the lower surface tension of these solvents can help prevent large forces from being applied to the suspended membranes while the solvent evaporates (either under ambient conditions or with careful use of forced nitrogen). Depending on the size of the starting Si wafer, typically several dozen 1cm×1cm chips supporting the Si-N platforms can be produced, with reasonably high yield. These are separated from the wafer by dicing or cleaving, and ready for background measurements or deposition of samples through appropriate shadow masks.

The dry-etch process must be used when either the sample (if put in place before the membrane patterning), thermometry, or other elements of the eventual device design will be attacked by the KOH or TMAH. Under such constraints, all the metal features and the windows in the Si-N can be pre-patterned on the front side of a double-side polished Si-N coated Si wafer, often with an additional Si-O${\;}_{x}$ layer added between the Si-N and Si. Via optical lithography using back-side alignment, a square window is then etched again via RIE. A thick photoresist layer is then patterned on the back of the wafer and the same window opened. The bulk Si is then etched via a two-step RIE Bosch process, where etch steps are alternated with a step that applies a passivating polymer to the Si sidewall. This Bosch process [70] allows very high aspect ratio trenches to be formed. The steps are alternated until the Si wafer is completely removed from the exposed areas. Here, the addition of the SiO${\;}_{x}$ layer helps improve yield since the Bosch process gases typically etch Si-N more rapidly than SiO${\;}_{x}$. The remaining SiO${\;}_{x}$ can be removed, if desired, with acid etch or another plasma process. Since the front side features are normally protected from the plasma etch using a resist or other removable protection layer, they are preserved as deposited, and the completed Si-N membrane structures are ready for use after removal of this protective layer.

## 3. In-plane thermal conductivity and Wiedemann–Franz Law

We begin a review of measurements of magnetic films or devices enabled by Si-N membrane thermal isolation platforms with a discussion of in-plane thermal conductivity measurements and a comparison with expectations of the Wiedemann–Franz law. Examples of Si-N platforms designed for such measurements are shown in Figure 1(c-e). Focusing on the structure shown in Figure 1(c-d), we see a platform composed of an 88×2050 μm${\;}^{2}$ bridge supported between two islands. Each island holds separate resistive heaters and thermometers (with 4-wire connections to each) as well as large triangular pads that provide electrical contacts to a sample film deposited on the bridge. Measurements with such platforms are carried out in vacuum of $10^{-5}$ Torr or better, with the platform clamped to a radiation-shielded gold-plated high-conductivity copper sample mount. The thermometers are calibrated in each experimental run against a reference thermometer mounted on the cryostat cold finger. The platform shown here uses microfabricated platinum thermometers, which maintain good sensitivity from well above room temperature to as low as 20 K or slightly lower.

**Figure 3.**
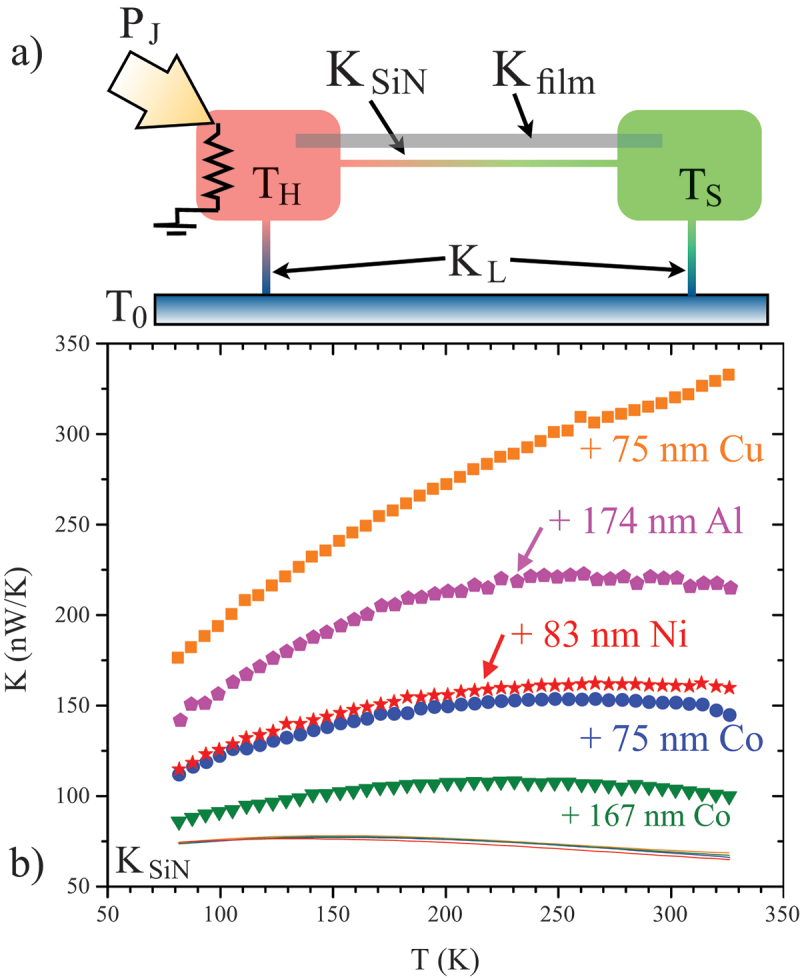
(a) Schematic view of the simple thermal model used to analyze heat flow in a two-island Si-N membrane thermal platform for measurements of film thermal conductance, $K_{\mathrm{film}}$. (b) $K_{\mathrm{film}}$ for several samples with thickness as noted, compared to two examples of the background thermal conductance of the Si-N beam connecting the two islands, $K_{\mathrm{SiN}}$ [29].

The thermal model that describes the platform is shown in Figure 3(a). This is used to understand the temperature differences generated when various levels of power are applied to the micromachined heater via Joule heating driven by application of a fixed current. The heat flow through the thermal isolation platform is dominated by conduction, since the relatively small area of the island on which the heating power is dissipated eliminates the radiation contribution typically problematic over 100K in DC thermal measurements and vacuum eliminates convection. Note that we do not include interfacial thermal resistances in this model, since the area of contact between the sample film and the even larger contact features on each island is large enough to make any interfacial contact resistance negligible compared to the much larger thermal resistance of the quite long Si-N bridge/sample structure. To be specific, if we take a typical value of thermal interface resistance for metal/oxide interfaces of $\approx 150\;\mathrm{MW}/Km^{2}$ [71] as an approximation to the metal/Si-N interfaces involved in the out-of-plane thermalization of the sample and contact pads on each island, considering that the area of contact is on the order of 90mm×90mm, the interfacial conductance is >1W/K, which is approximately 8 orders of magnitude larger than the thermal conductance of the Si-N bridge and sample. This shows that, while interfacial thermal conductance can be an important consideration for nanostructures, as shown in Figure 1(a, f), it need not be considered for the larger structures designed for measurements of thin films. Assuming a steady state and a small signal limit, this model can be solved to yield expressions for $K_{B}=K_{\mathrm{Si}-N}+K_{\mathrm{Film}}$, the thermal conductance of the bridge, and $K_{L}$, the conductance of the supporting legs, as a function of P, which is the measured power dissipated in the sample heater, such that:

(1) $$T_{H}=T_{o}+\left[\frac{\left(K_{L}+K_{B}\right)}{(2K_{B}+K_{L})K_{L}}\right]P,$$

(2) $$T_{S}=T_{o}+\left[\frac{K_{B}}{(2K_{B}+K_{L})K_{L}}\right]P.$$

We then determine $K_{B}$ and $K_{L}$ from the measured slopes of ΔT vs. P for each island, which as seen above for a pair of equations with $K_{B}$ and $K_{L}$ the only unknowns [27–29]. We determine the contribution of a thin-film sample by first measuring $K_{B}$ for the bare Si-N bridge (as shown in solid lines in Figure 3(b), and subtracting this background from $K_{B}$ measured again after the film is deposited. These values of total conductance are much larger than $K_{\mathrm{Si}-N}$ even for very thin metal films, as shown for Al, Cu, Ni, and Co. Previous examinations have shown that Si-N membranes fabricated on the same wafer show a relatively high degree of uniformity, but with more variability than expected from the geometry of the beam, which is typically very uniform using photolithographic patterning [28]. This variability is small compared to the contribution to K from many thin metal films, but when small sample thermal conductance is expected, the best practice is often to pre-measure the exact bridge structure before adding the sample film. k for the film is determined from the film contribution using the lateral geometry of the Si-N bridge and the thickness of the sample determined via profilometry on a separate witness sample.

Resistance measurements of the sample deposited on the platform are straightforward and allow for determination of the Wiedemann–Franz ratio, $L=k/\mathrm{\sigma T}=K_{\mathrm{film}}R_{\mathrm{film}}/T$. Here, the common sample geometry cancels, leaving only extensive transport properties. This removes the often dominant source of error from the determination of L. Estimated combined statistical and systematic error on L is $2\times 10^{-10}\;W\Omega /K^{2}$ or better. Surface-scattering induced modification of the Si-N background K can introduce systematic errors where $K_{\mathrm{film}}$ is unusually small. Measures to understand and manage these surface scattering effects, when needed, have been described [28,29].

In a conducting material, one normally expects the ratio of electrical and thermal conductivity to follow the Wiedemann–Franz (WF) law, k/σ=LT, since the physics of charge transport in a Fermi liquid is typically robust. However, deviations from this relation are well known and can occur even in bulk crystals when the scattering of electrons by phonons with moderate wavefunction, q, removes kinetic energy from the conduction electron without changing its direction. Such ‘vertical’ scattering processes are relatively frequent in a temperature range below the Debye temperature but above the disorder scattering limit [72]. Scattering from large q phonons and from static disorder, defects, or imperfections tends to affect both energy and charge carrying processes of electrons in the same way, which means such scattering events maintain the WF law prediction. Overall, bulk materials do show some dependence of L on material and temperature and often deviate somewhat from the free-electron Sommerfeld value of $L_{0}=2.44\times 10^{-8}\;\mathrm{WW}/K^{2}$ [73].

In thin films and nanostructures, a somewhat complicated picture of the WF law has now emerged, which is perhaps not surprising considering the wider range of more frequent electron scattering effects one typically finds in these systems. Recent reports include ‘violations’ of the WF law in fairly exotic systems [74–79] and simple metals with reduced dimensions [80–86], and also confirmation of WF behavior at the scale of atomic junctions [87,88] and in certain thin films and nanowires [68,89]. Below we review recent progress in this area enabled by Si-N membrane thermal platforms.

**Figure 4.**
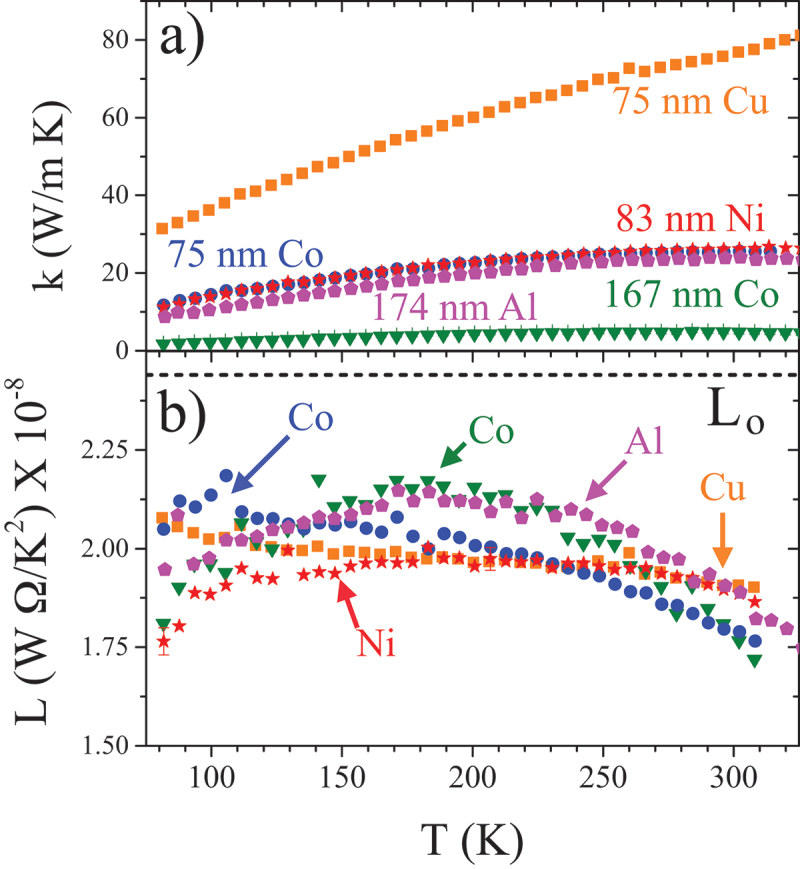
(a) k vs. T for the same range of films shown in fig. 3. (b) Measured L for these films compared to the free-electron value, $L_{0}$. All films fall in a similar range, and all reduced from $L_{0}$ with a slight T dependence [29].

Figs. 4a) and b) show k and L, respectively, measured for Al, Cu, Ni, and two Co films. Note that the thicker Co film was grown under far-from-ideal UHV conditions and contains a large number of static defects. In Figure 4(b), the free-electron Sommerfeld value $L_{o}$ is also shown (dashed line).

The measured L for all five films show similar values and behavior with T, and all are depressed from $L_{o}$, which is not uncommon for simple polycrystalline metal films [29]. This is not universally true; however, as evaporated gold films show an interesting and not yet completely understood thickness-dependent strong reduction in k compared to expectations of the Wiedemann-Franz law [86].

**Figure 5.**
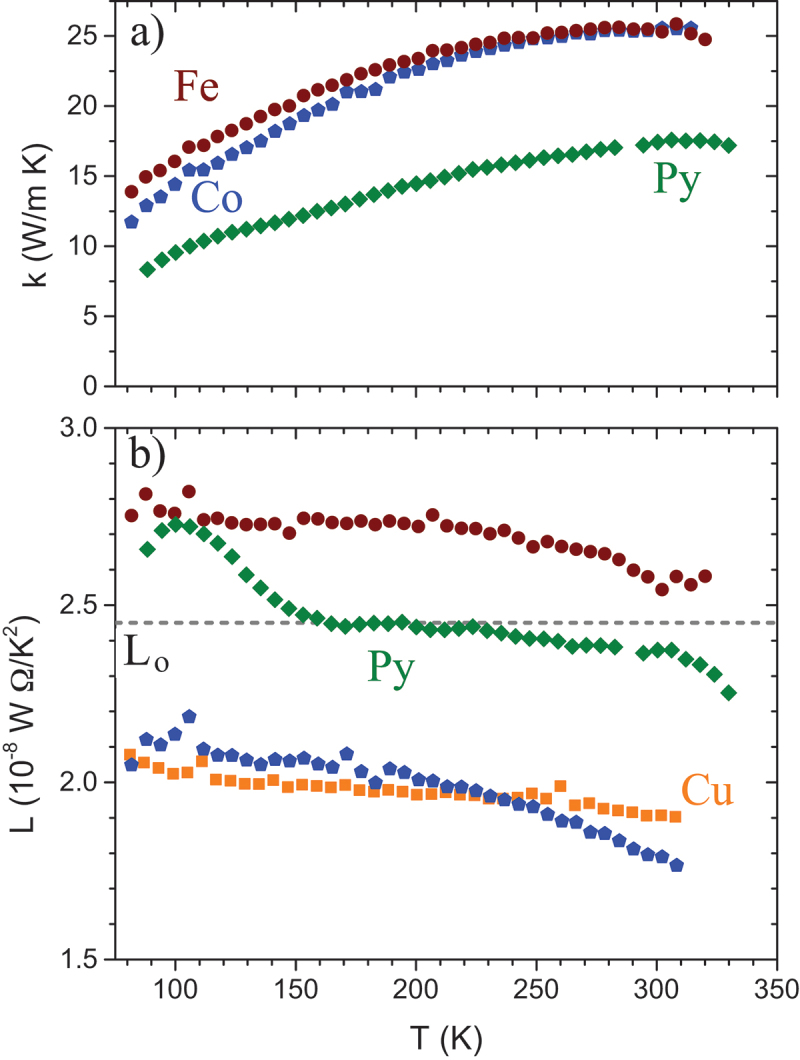
(a) k vs. T for three thin films of 3d transition metal ferromagnets, a 75 nm Co film, a 54 nm permalloy (Py) film [29] and a 75 nm Fe film [55]. The Ni-Fe alloy permalloy film has a lower k, as expected due to higher disorder scattering of the electrons that dominate its thermal conductivity. (b) Measured Lorenz number L vs. T for the same films, compared also to the 75 nm Cu film. The Fe film somewhat exceeds $L_{0}$, while Co is similar to Cu films in showing slight reductions in k compared to expectations based on its electrical conductivity. The disorder scattering, which introduces additional high-q electron-defect scattering events, gives the best match to the Wiedemann–Franz prediction that has been observed using Si-N membrane thermal platforms.

Figure 5 summarizes similar results with a focus on metallic ferromagnets of interest for a range of spintronic devices. Figure 5(a) compares k for three examples of 3d transition metal ferromagnets, a 75 nm Fe film [55], a 75 nm Co film, and a 56 nm permalloy (the Ni-Fe alloy typically with 80% Ni) film [29]. Bulk values of k for Fe and Co are both much larger throughout this temperature range, at 80 and 90W/mK at 300 K [90], in line with the expectation of limits on heat conduction from scattering of electrons (or other heat carriers) from grain boundaries, surfaces, and other defects. Additional disorder scattering explains the further reduction of k in the alloy film, which is well below the single-component films at all T. The exact nature of the electron scattering in the film has a stronger influence on measured L as shown in Figure 5(b). Here, as shown also in Figure 4(b), the Co film has the same slight reduction as seen in Cu, while the best agreement with the WF law using $L_{0}$ occurs in the alloy film, due to the additional disorder scattering, which introduces additional high-q electron-defect scattering events that affect thermal and charge transport processes in the same fashion [29]. Data for Fe fall significantly above $L_{0}$, which could indicate additional thermal conductivity contributions other than electrons. A detailed understanding of this contribution in Fe is not yet available, though theoretical predictions of magnon conductivity in bulk and single crystal iron have emerged in recent years [91,92]. The non-electronic contributions to k seen in Fe and absent in the other 3d ferromagnets shown here are likely related to the same dominance of spin-disorder scattering of electrons at the Fermi level in Fe that drives the emergence of a strong magnon drag thermopower (as discussed further below) compared to other 3d ferromagnets where s−d scattering plays a larger role [93].

**Figure 6.**
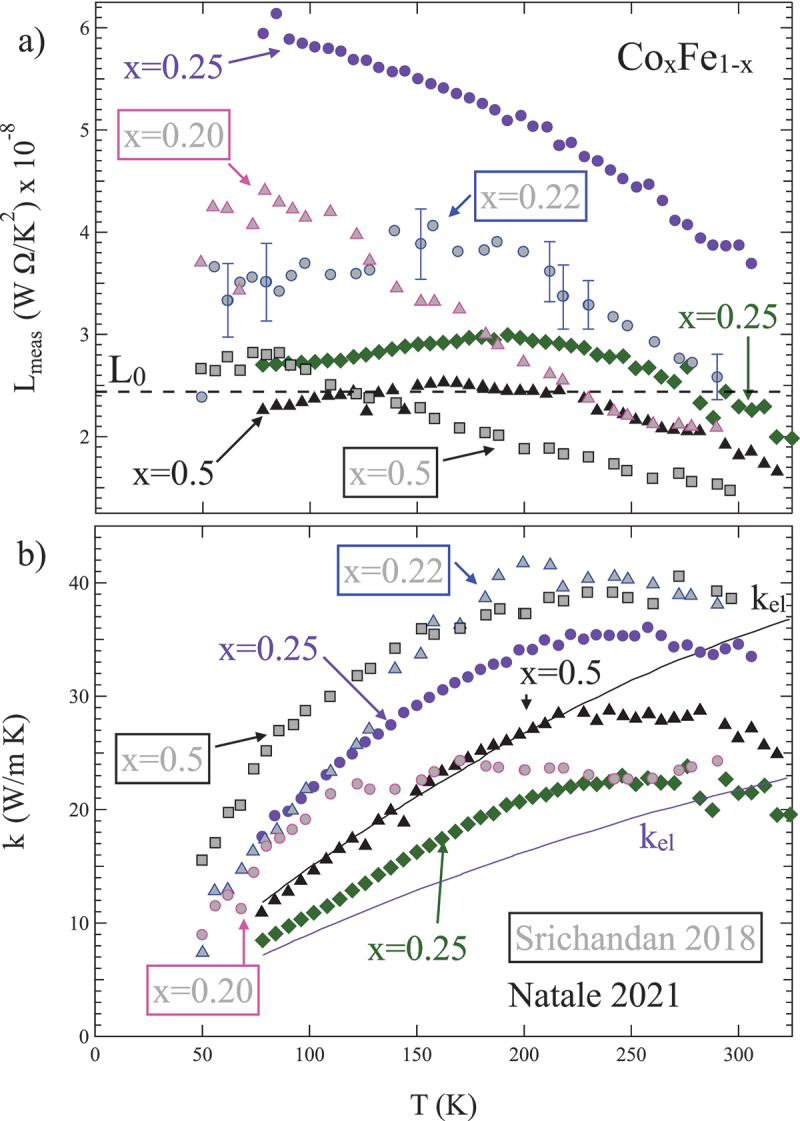
Review of k for sputtered Co${\;}_{x}$Fe${\;}_{1-x}$ films measured using two types of Si-N membrane thermal platform. In both plots, grey-filled symbols are data from Srichandan, *et al*. [94] and solid symbols are from Natale *et al*. [95] (a) measured Lorenz number, $k_{\mathrm{film}}/\sigma_{\mathrm{film}}T$, vs. T compared to the free-electron value $L_{0}$ (dashed black line). Data falling above $L_{0}$ is often taken as evidence of non-electronic contributions to k. Co${\;}_{x}$Fe${\;}_{1-x}$ films near the composition dependent minimum in Gilbert damping at x=0.25 [96] have $L_{\mathrm{meas}}>L_{0}$ for most T. (b) Corresponding k vs. T for the same set of films. Here, the predicted electronic k is also shown for two of the films from Natale, *et al*., the solid black line which matches the measured k for most T for the relatively high damping x=0.5 film, and the solid magenta line, which gives predicted electronic k for the low damping x=0.25 films, which falls well below $k_{\mathrm{meas}}$ for both x=0.25 films in that work.

Figure 6 reviews the current experimental knowledge for the special case of cobalt-iron alloys, Co${\;}_{x}$Fe${\;}_{1-x}$, a material which has among the lowest known Gilbert damping parameters for a ferromagnetic metal [96–99]. This means that spin excitations, such as magnons or spin waves, have much longer lifetimes in this material than in typical 3d transition metal ferromagnetic thin films such as Fe, Co, or Py. This damping parameter depends on composition, such that alloys with Co fraction near x=25 have much lower damping parameter than alloys with either much higher or lower Co fraction, including alloys with x∼50 or higher. Figure 6(a) reviews experimental evidence from two groups for large values of measured L, noted $L_{\mathrm{meas}}$ here, vs. T for a range of Co${\;}_{x}$Fe${\;}_{1-x}$ films measured with Si-N membrane platforms [94,95]. Again, the free-electron value $L_{0}$ is indicated with a dashed line. Outlined symbols with grey interior are taken from Srichandan, *et al*. [94] and solid symbols from Natale, *et al*. [95]. Both groups reported values for x=0.5 films, which fall near or below $L_{0}$ for most of the measured T range. In rather stark contrast, compositions near the x=0.25 minimum in damping show strong positive contributions to $L_{\mathrm{meas}}$. Note that Natale *et al*. reported values for two films with nominally equal compositions (grown in the same sputtering run), which had rather different $L_{\mathrm{meas}}$, suggesting a strong dependence on small variations in composition [95].

Figure 6(b) presents k vs. T for the same selection of films. Since the electrical conductivity of these alloys also changes with x, this view is somewhat more complicated, since the details of each group’s growth process result in variations in film morphology that affects both σ and k. Note also that the estimated purely electronic contribution to k, $k_{\mathrm{el}}$, as determined from the WF law, is shown for the Natale, *et al*. data for both x=0.5, which matches the measured k data well for much of the T range, and one x=0.25 sample (the second had a similar $k_{\mathrm{el}}$) [95].

Despite the very similar phenomenology of these two studies, the two groups interpreted the excess k somewhat differently, with Natale *et al*. suggesting a role for spin dynamics in the form of magnons or spin waves, while Srichandan did not envoke magnons. However, Natale *et al*.’s study also included a clear demonstration of a magnetic field direction dependent k in the low damping x=25 sample that is totally absent in the x=50 sample, which is at the very least suggestive of the role of magnon thermal conductivity in this ferromagnetic metal. Additional experiments are required to resolve these issues, and work along these lines is progressing.

## 4. Seebeck coefficient, Peltier effect, and Magnon Drag

**Figure 7.**
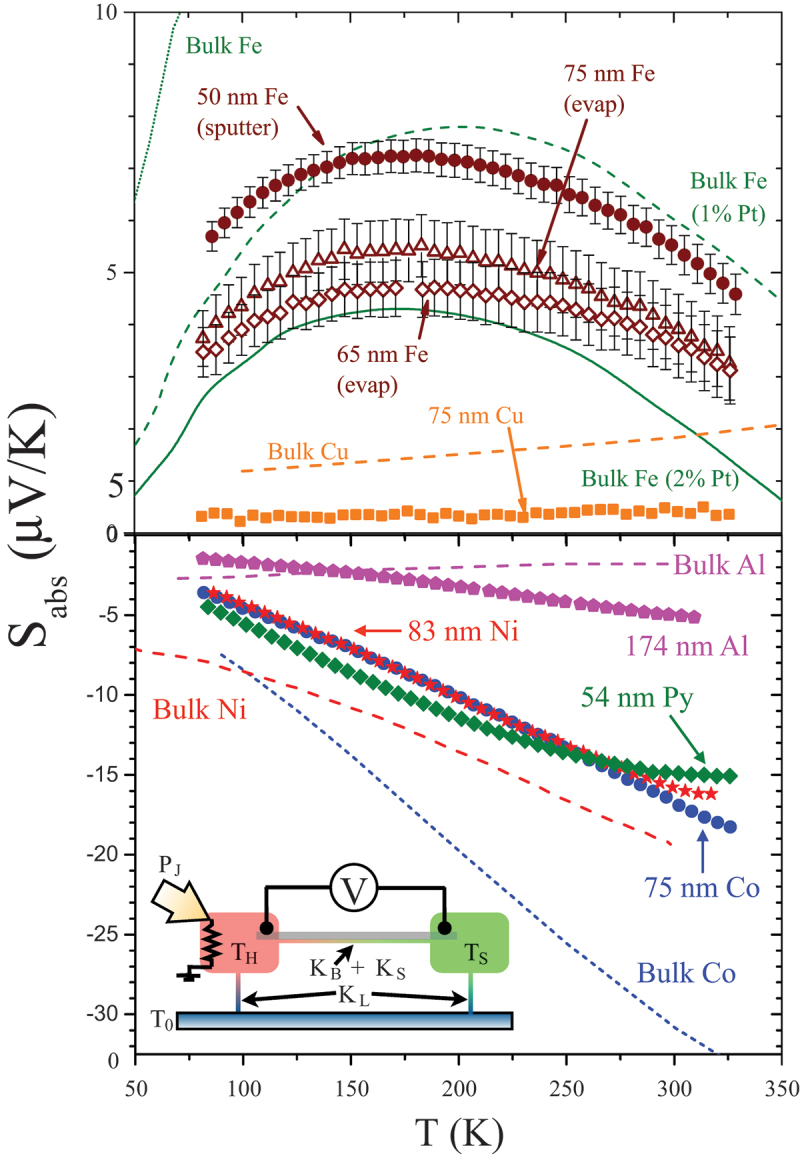
$S_{\mathrm{abs}}$ vs. T for a range of FM metal films, as well as selected non magnetic films and bulk materials. Note the positive and negative ranges are on different scales. Based on this (and other) data for thin films, the general expectation is that the Seebeck coefficient is typically lower for a film compared to bulk, which is likely related to contributions from defects and grain boundaries. *Inset*: Schematic view of the thermopower experiment, where leads at the ends of the sample are used to measure longitudinal voltage while an in-plane temperature gradient is applied by heating one island in a device, as shown in fig. 1c) or e).

In addition to the quantitative examination of electronic and other contributions to thermal conductivity, the Si-N membrane thermal platform's ability to measure electrical signals from a thin film while a controlled and well-understood thermal gradient is applied is powerful for probing a range of thermoelectric and magnetothermoelectric effects. The first of these to be overviewed is the Seebeck coefficient, or longitudinal thermopower. This arises since mobile charge carriers in a material where a thermal gradient exists will redistribute, causing a flow of charge carriers in steady state under a thermal gradient in equilibrium where carriers flow from hot to cold. In a semiconductor with only one type of charge carrier, the sign of the resulting voltage measured at the ends of a sample as shown schematically in the inset of Figure 7 can indicate the type of carrier. In a metal, either sign of the thermal voltage (or thermopower) can occur, along with near zero thermopower, and this depends on the slope of the density of states at the Fermi level. This range of sign and size of thermopower, or Seebeck coefficient, occurs both in bulk metals and in thin films, though the now growing but still somewhat limited body of Seebeck coefficient measurements for thin films shows that, in general, the Seebeck coefficient is reduced in films compared to values for the corresponding bulk material. This is perhaps not tremendously surprising, since even in bulk materials, impurities, vacancies, and size effects all contribute to thermopower, and can certainly work to reduce the total magnitude of the thermopower [100–104]. All these imperfections should generally be expected to occur frequently even in high-quality thin films.

In any experiment to measure thermopower, where voltage leads are attached to the hot and cold sides of a sample under an applied thermal gradient, the thermovoltage has contributions from both the sample and the leads that complete the circuit, such that the thermopower defined by dividing the thermal voltage by the temperature difference gives what is called the relative thermopower, $S_{\mathrm{rel}}=V/\mathrm{\Delta T}=S_{\mathrm{abs}}-S_{\mathrm{leads}}$. Determining the absolute thermopower, $S_{\mathrm{abs}}$, which gives information exclusively about the sample, requires separate methods to determine the lead contributions. Experiments and methods to achieve this for Si-N thermal isolation platforms are described elsewhere [105]. The data shown here have been corrected for the lead contribution using those methods.

Figure 7 presents $S_{\mathrm{abs}}$ vs. T for a range of thin films with a focus on metallic ferromagnets or other materials relevant to spintronic devices. This figure includes both negative and positive $S_{\mathrm{abs}}$, and compares values for films to selected bulk materials. The largest positive values shown here are for three Fe films, with various thickness and preparation techniques. Despite some shift in the absolute magnitude of these curves, they all show a similar broad peak near 200 K, which also appears in nominally pure bulk Fe (not fully visible in Figure 7) and in dilute bulk alloys of Fe with Pt [93]. This peak is typically associated with interactions between magnons and conduction electrons, termed magnon drag, in analogy to a similar interaction between phonons and electrons termed phonon drag [106,107]. In both effects, momentum is transferred to conduction electrons by quasiparticles driven by the thermal gradient, therefore adding to the thermopower, usually with a characteristic temperature dependence. Phonon drag is typically seen near the peak in a bulk sample’s thermal conductivity and is driven by the temperature-dependence of the phonon relaxation times. The main phenomenological distinction between phonon and magnon drag is that the phonon drag effect is very strongly quenched by disorder, while magnon effects are less sensitive to the material imperfections that quickly limit the phonon lifetime in real materials. A much smaller positive $S_{\mathrm{abs}}$ is seen in a thin film Cu sample, and again, this is reduced compared to values for bulk Cu (orange dashed line) [108]. A somewhat thicker aluminum thin film has a modest negative $S_{\mathrm{abs}}$ which is of similar magnitude to bulk values [109] but has a different slope. Ni, Co, and permalloy thin films all have quite similar absolute Seebeck coefficients and have significant negative values, as also seen in the bulk. The large values of the Seebeck coefficient are part of the motivation to use these metals in energy harvesting technologies. Again, the values for these films are reduced from tabulated bulk values [93,110].

Note also that most of these films are predominantly linear as a function of T. One can understand this using the common theory relating the resistivity of a conductor to its absolute thermopower. When mobile charges flow from hot to cold via diffusion, the resulting Seebeck coefficient can be explained using the Mott equation, which is typically written for metals as:

(3) $$\alpha_{\mathrm{Mott}}=-\frac{\pi^{2}k_{B}^{2}T}{3e}\frac{1}{\rho \left(E\right)}{\left[\frac{\partial \rho \left(E\right)}{\mathrm{\partial E}}\right]}_{E=E_{F}},$$

where ρ is the electrical resistivity, $E_{F}$ is the energy at the Fermi level, $k_{B}$ is the Boltzmann constant, T is temperature, e the charge of the carrier, and $[\partial \rho /\mathrm{\partial E}]{|}_{E_{F}}$ is the energy derivative of the resistivity evaluated at the Fermi energy. Here, the only explicit T dependence is linear, though if the energy dependence of the resistivity (1/conductivity) has any T dependence this will cause a somewhat nonlinear diffusive thermopower.

Si-N membrane thermal platforms also enable a fairly rare ability to directly measure the Peltier effect, which is the Onsager reciprocal effect to the standard longitudinal thermopower [111]. Here, the current is driven through the thin-film sample, and the resulting temperature difference measured with the calibrated thermometers on each island [112]. Results of these experiments for typical transition metal FM thin films confirm the prediction of Onsager reciprocity in magnetic field-independent measurements as a function of T. At the time, the measurement technique was not sensitive enough to detect the sign change in the Peltier coefficient that Onsager reciprocity predicts due to broken time-reversal symmetry, and this remains an opportunity for future work.

**Figure 8.**
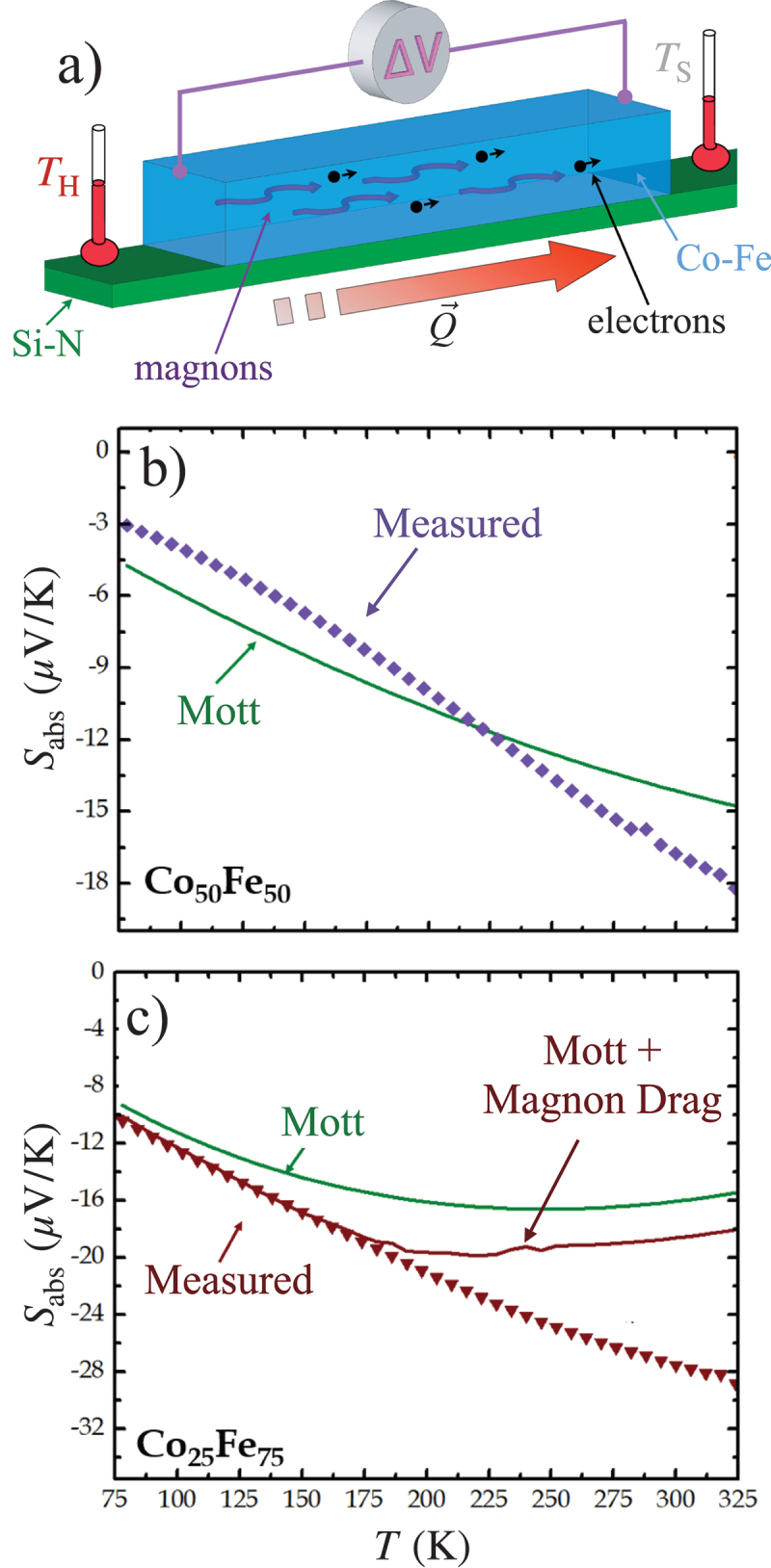
(a) Schematic view of magnon drag thermopower. The magnons or spin waves in the low-damping Co-Fe material transfer additional momentum to the conduction electrons, increasing the voltage measured at a given applied temperature difference. (b) Comparison of the estimated diffusive contribution (labeled ‘Mott’) and measured $S_{\mathrm{abs}}$ for the Co${\;}_{50}$Fe${\;}_{50}$, which has damping typical of a 3d transition metal FM. c) Similar comparison for the low-damping Co${\;}_{25}$Fe${\;}_{75}$ composition, where the Mott equation predicts a diffusive component much less than measured $S_{\mathrm{abs}}$. Adding a theoretical prediction for the magnon drag to diffusive thermopower achieves excellent agreement at the lowest measured T [113].

Magnon drag, shown schematically in Fig. 8(a), is apparent in Fe, and not visible at these temperatures for Ni or Co, due to a balance between reasonably low Gilbert damping in Fe, while maintaining enough coupling between magnons and conduction electrons [93,114]. This balance is also realized in the Co${\;}_{x}$Fe${\;}_{1-x}$ alloys [94,113,115], where the composition-dependent Gilbert damping causes a significantly larger magnitude of $S_{\mathrm{abs}}$ for compositions near x=0.25. Example data for two selected samples are shown in Figure 8(b, c) [113]. As was the case with thermal conductivity, when the low damping parameter of the x=0.25 material enables longer lived magnons, the stronger drag effect causes a larger negative $S_{\mathrm{abs}}$ than seen in the x=0.5 sample that shows more typical damping. Though demonstrating the qualitative existence of magnon drag in this way is often relatively straightforward, quantitatively separating the magnon effects from diffusive thermopower terms is typically very challenging. For example, one can consider the Mott equation (Eq. 3) as a route to predict diffusive thermopower, but the use of this form requires some knowledge of the dependence of electrical resistivity on the energy of the conduction electrons, which is not at all simple to determine from typical transport experiments. In certain cases, the magnetic field dependence of the thermpower and the associated anisotropic magnetoresistance can allow for an estimate of this energy dependence [113,116]. Using these techniques to estimate dρ/dE allows for reasonable prediction of the diffusion thermopower, as plotted in solid lines in Figure 8(b, c). The resulting estimate is never more than several μV/K away from the measured $S_{\mathrm{abs}}$ for the higher damping x=0.5 film, while it falls far short of explaining the measured $S_{\mathrm{abs}}$ for x=0.25. When a theoretical calculation of the magnon drag contribution is added to the estimated diffusion thermopower, an excellent match to the measured values is seen in the low T limit [113]. Finally, note that the magnitude of $S_{\mathrm{abs}}$ is largest for the Co${\;}_{25}$Fe${\;}_{75}$ sample than for any other ferromagnetic films studied with Si-N membrane techniques.

## 5. Anomalous Nernst effect, planar Nernst effect

Quantifying a range of transverse thermopowers, where the voltage is measured orthogonal to an applied thermal gradient, has also proven to be an important area for Si-N membrane thermal platforms. Here, a much clearer understanding of the direction of the thermal gradient is a huge advantage for clarifying the physics of a material or device under investigation. The ability to use simple modeling, due to the essentially 2d nature of the heat flow in a Si-N membrane structure [30,36,116–119] also provides an important advantage for these techniques. Here, I focus on two particularly important areas where Si-N membranes clarify the physics of these transverse effects, generation of anomalous Nernst effect contributions to nanoscale metallic non-local spin valves and measurements of the planar Nernst effect in metallic conductors.

**Figure 9.**
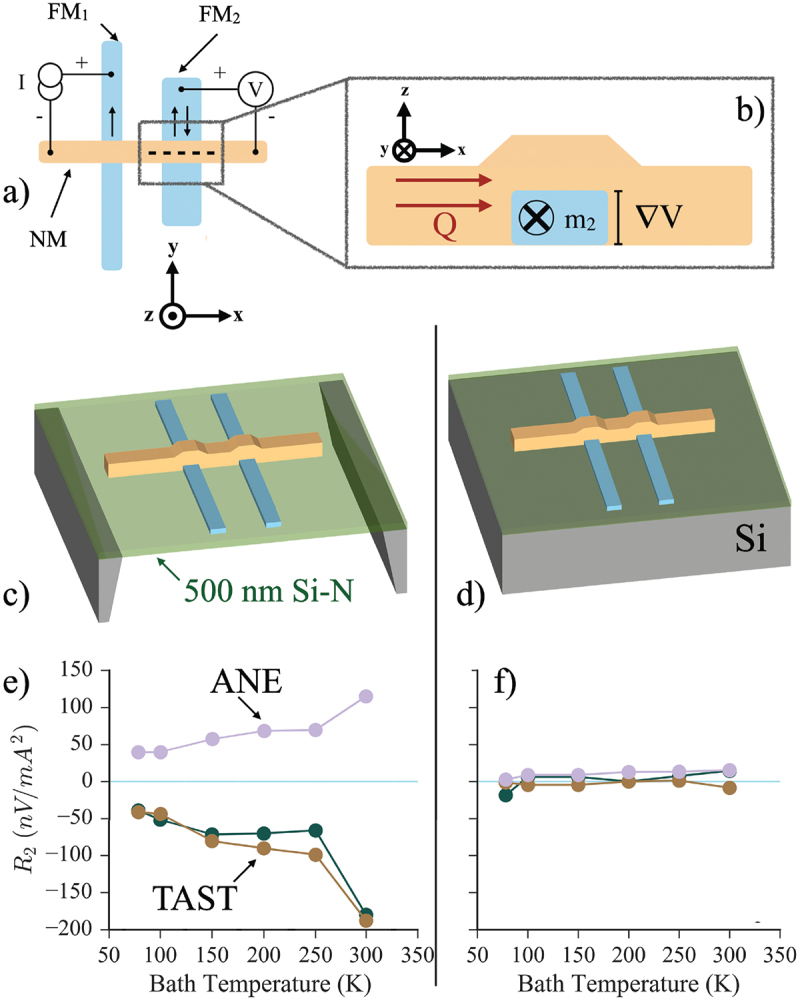
(a) Schematic view of a nanoscale metallic non local spin valve, where two ferromagnetic nanowires separated by a distance L are bridged by a nonmagnetic (NM) channel (typically made of Cu or Al). The magnetic field dependence of the voltage measured between FM${\;}_{2}$ when a current is driven from FM${\;}_{1}$ to the NM channel provides a measure of the spin accumulation injected into the NM channel. (b) Cross-sectional view of FM${\;}_{2}$, where in-plane heat flow Q drives a thermal gradient ∇T in the $\hat{x}$ direction as defined in the figure. Since the magnetization of FM${\;}_{2}$ is in the $\hat{y}$ direction, the anomalous Nernst effect drives an electric field in the $\hat{z}$ direction, which leads to a measurable signal component in the nonlocal voltage. (c) Schematic view of the NLSV fabricated on a free-standing Si-N membrane. (d) A similar view of the NLSV fabricated on a supporting bulk substrate, which leads to very different thermal gradients when the NLSV is operated. (e) Thermal component of the non-local resistance vs T for a membrane-supported NLSV. Purple data shows the field conditions selected to isolate contributions from the anomalous Nernst effect, while green and orange data isolate the thermal contributions to spin transport. (f) The same plot for the substrate-supported NLSV, where all thermally driven signals are dramatically reduced.

The metallic non-local spin valve (NLSV) is a spintronic device used to generate and study pure spin currents [120–128], which could also soon see industrial applications as a read-head in magnetic recording systems [129–133]. Generation of thermal gradients plays an important role in these devices, and thermal effects have been known for at least a decade to affect the background resistance [134–138], lead to signals through various thermomagnetic effects [139], and offer the possibility to generate spin in NLSVs [140–149]. Figure 9(a-b) show the NLSV schematically and overview how signals due to the anomalous Nernst effect (ANE) can be generated in these structures. The NLSV is formed from two FM nanowires separated by a distance on the order of the spin diffusion length in the material that forms the non-magnetic channel. When the electrical current is driven from the left FM through the left side of the NM channel, the spin-polarized current injects non-equilibrium angular momentum into the NM channel along with the charge. This accumulation of non-equilibrium spin diffuses through the channel, such that the voltage measured between the right FM and the right side of the channel depends on the in-plane magnetic field applied in the $\hat{y}$ direction as indicated, and this voltage probes the value of the spin current that diffuses through the channel.

The ANE is the thermoelectric analog to the anomalous Hall effect [4,150–153]. Several definitions of this effect have been used in relevant recent literature. Here I follow the convention used by Slachter, *et al*. [139], since the main use of Si-N membranes for ANE studies, to date, has involved non-local spin valves. The electric field produced by the ANE is given by the equation

(4) $$\nabla V_{N}=-S_{N}\hat{m}\times \nabla T$$

where $\nabla V_{N}$ is the electric field due to the ANE, $S_{N}=R_{N}S_{\mathrm{FM}}$ is the transverse Seebeck coefficient dependent on the Seebeck coefficient $S_{\mathrm{FM}}$ and the anomalous Nernst coefficient $R_{N}$ of the ferromagnetic material, $\hat{m}$ is the unit vector in the direction of the magnetization of the FM detector, and ∇T is the thermal gradient. Note that the expressions used by Chuang, *et al*. [151] and some other authors are equivalent, though they use the term ‘Anomalous Nernst Angle’ and the symbol $\theta_{N}$ where I used $R_{N}$. Some authors choose to write expressions more obviously tied to the Onsager formalism for thermoelectricity, such that

(5) $$S-S_{N}=\rho_{\mathrm{xx}}\alpha_{\mathrm{xy}}-\rho_{\mathrm{yx}}\alpha_{\mathrm{xx}},$$

where $\rho_{\mathrm{xx}}$ is the longitudinal resistivity, $\rho_{\mathrm{yx}}$ is the anomalous Hall resistivity, $\alpha_{\mathrm{xx}}$ is the longitudinal thermoelectric conductivity, and $\alpha_{\mathrm{xy}}$ is the transverse thermoelectric conductivity [154,155]. For metallic ferromagnets, the second term is nearly always tiny compared to the first, as has been demonstrated explicitly [154]. The remaining term can be expressed in terms of the longitudinal Seebeck coefficient [3], which leads to the expression in Eq. 4. However, when comparing data across publications, the author urges readers to confirm the details of measured quantities, confirm the sign convention used in defining $S_{N}$, and pay close attention to units.

Returning to the manifestation of ANE in non-local spin valves, Fig. 9(b) shows a cross-sectional view of the spin-detecting FM strip, where the heat that flows along the x-direction in the NM channel is shown. This heat flow generates a component of the in-plane thermal gradient at the location of the FM. This produces an electric field, and a measurable voltage, between the NM and the FM since these overlap at the location of the FM such that a portion of the electrical path is in the mutually orthogonal $\hat{z}$ direction. This produces a signal in the non-local voltage when measured as a function of the applied in-plane H that is antisymmetric, where the spin signal is symmetric with field [119,139,156].

The ability to fabricate the NLSV on a Si-N membrane allows for a dramatic change in the thermal gradient that generates ANE signals in the NLSV compared to a device fabricated on a bulk substrate. The two methods of fabrication are shown schematically in Fig. 9(c, d), with the corresponding thermal portion of the NSLV signals plotted vs T in Fig. 9(e, f). When supported on a membrane, applying the same driving current to a similar NLSV structure results in a much larger in-plane thermal gradient, which drives the ANE signal to much larger values than those seen in the substrate-supported NLSV. This makes membrane-supported NSLVs a relatively effective tool for quantifying ANE in the material forming one FM in an NLSV. In addition to the ANE, which can be separated from purely spin effects using the field-symmetry of the signal, the Si-N membrane-supported NLSV shows a previously unknown effect, where electrically injected spin transport is assisted by the presence of the in-plane thermal gradient. This signal is identified in Fig. 9(e, f) as thermally assisted spin transport (TAST), which also vanishes when the larger in-plane thermal gradient vanishes when the NLSV is placed on a bulk substrate.

**Figure 10.**
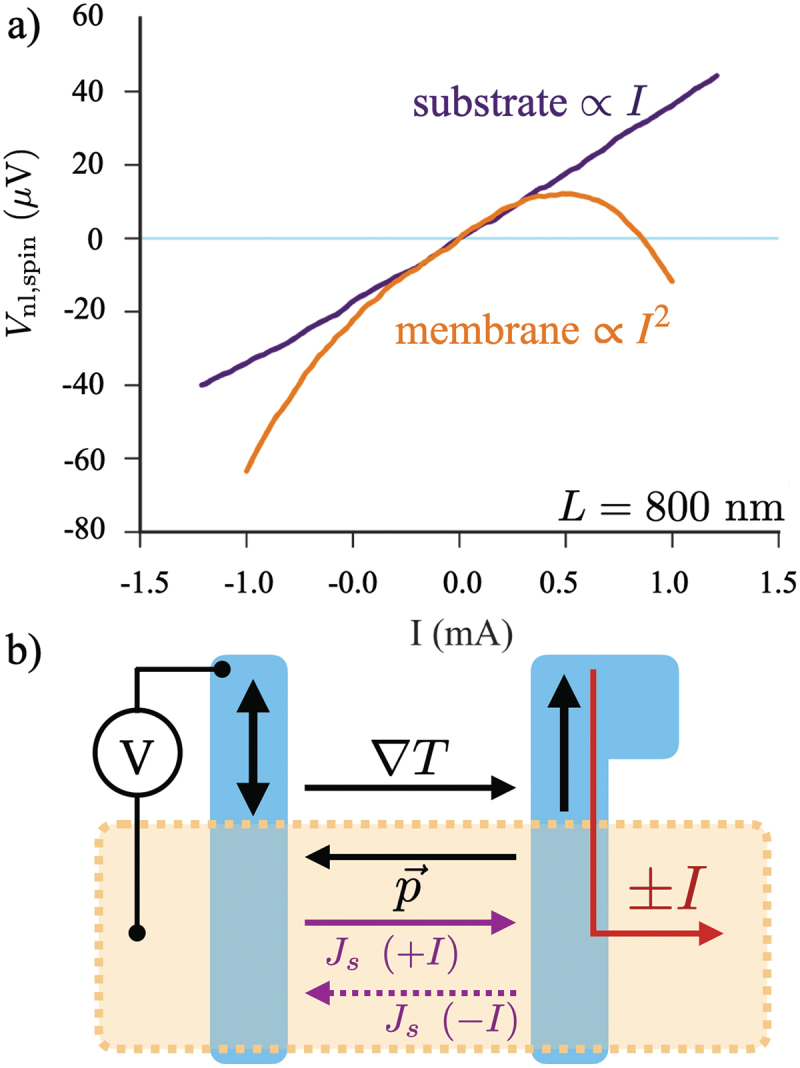
(a) Spin-selected non-local voltage $V_{\mathrm{nl},\mathrm{spin}}$ vs bias current I for two Py/al NLSVs fabricated on the same chip, but with one suspended on a membrane and the other supported by a bulk substrate. Data was taken at T=78K bath temperature for both. The two have the same slope for low I, before a large in-plane thermal gradient develops in the Si-N membrane supported NLSV, which interacts with the electrically injected spin current. (b) Schematic view of the potential physics of this thermally assisted spin transport. The flow of phonons driven by the in-plane thermal gradient transfers momentum to spins, assisting the spin current flow in one orientation (indicated by the dashed arrow labeled $J_{s}(-I)$) and resisting it in the other (indicated by the solid arrow labeled $J_{s}(+I)$ [119].

Figure 10(a) shows a specific example of this effect, demonstrated by comparing the IV characteristics for two similar NLSVs with separation L=800nm for a membrane-supported device and a substrate-supported one. Here, the background thermal effects and the contribution from the ANE are by measuring the full IV curve, here using a modified dc current-reversal technique, at two magnetic fields selected to isolate pure spin effects, and subtracting them [119], and this isolated pure spin voltage $V_{\mathrm{nl},\mathrm{spin}}$ is then plotted vs I. The slope of the $I-V_{\mathrm{nl},\mathrm{spin}}$ curve is the typical spin resistance, and the linear behavior of such a plot indicates that electrical spin injection dominates the response of the NLSV, while the non-linear behavior indicates thermally driven signal components [147,157]. Here, both NLSVs have the same spin resistance for low values of I, but as the bias increases, the behavior of the membrane-supported device differs. At large negative current, the spin-dependent voltage (with field selected to eliminate the ANE contribution) grows more negative, indicating a thermal effect that drives further spin current down the channel. At large positive current, the heating in the membrane NLSV now reduces the spin flow, eventually driving $V_{\mathrm{nl},\mathrm{spin}}$ negative, indicating a reversal in the spin current flow in the channel due to the heating. This interaction of the in-plane thermal gradient in the Si-N membrane supported NLSV is shown schematically in Fig. 10(b). The current bias heats the injector FM (shown here at left), driving the large in-plane gradient ∇T. This leads to a flow of phonons in the channel, which has an overall average additional momentum indicated by vector $\vec{p}$. This is driven by ∇T and is independent of the sign of the current. However, the direction of electrically injected spin current flow in the device does depend on the sign of the current. This phonon-driven momentum assists the spin current when I is negative and resists it when I is negative, as shown by the purple vectors. Further work is needed to understand this thermally assisted spin transport in detail.

**Figure 11.**
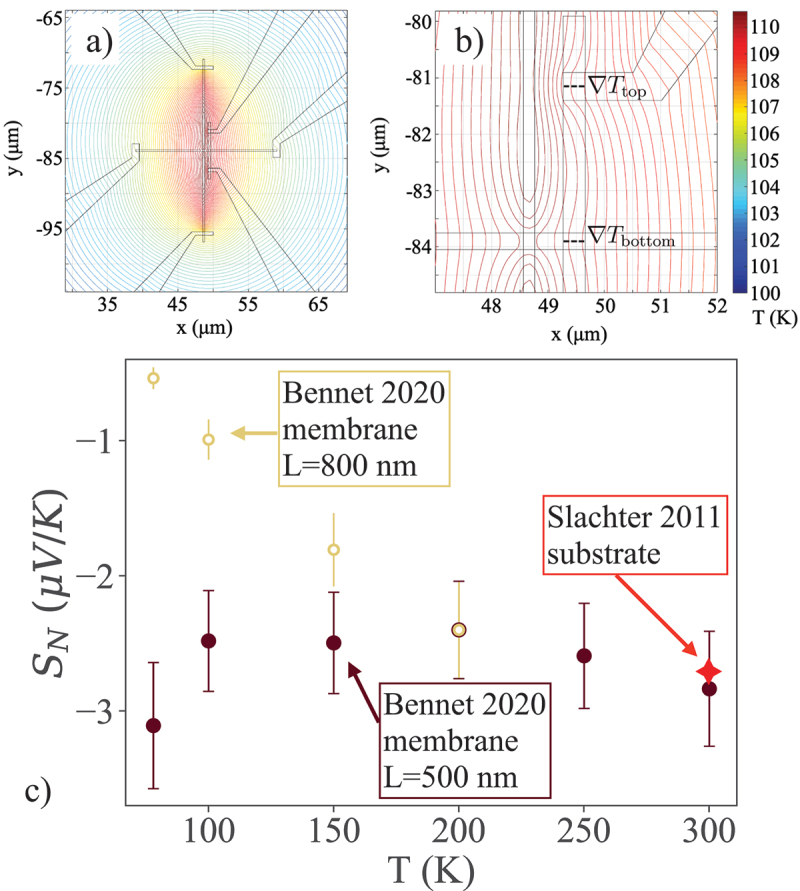
(a) 2d finite element thermal model prediction of the temperature of a Si-N membrane supported NLSV during operation (here where current is driven exclusively through the injector FM) [156]. (b) A closer view of the active NLSV area shows not only that the entire device heats but also that in-plane thermal gradients exist at both locations where the detector FM is crossed by Al wires, one that forms the spin channel and the second that makes the voltage connection. Both must be taken into account to determine the ANE contribution [119,156]. (c) The resulting transverse Seebeck coefficient, $S_{N}$, from two Si-N membrane supported NLSVs vs T [156], compared to the value for a substrate-supported NLSV [139].

The ability to simplify thermal modeling in the Si-N membrane supported NSLV, along with the determination of a more appropriate choice of the thin-film material property inputs to thermal modeling allowed by k and $S_{\mathrm{abs}}$ measurements using Si-N membrane thermal platforms, enables careful examination of the ANE for the FM forming the NLSV. Figure 11 overviews an example of this procedure, with panels a) and b) showing two views of the thermal profile calculated from 2d finite element modeling of the Si-N membrane-supported NLSV. One important insight from this modeling is that ANE contributions occur in the membrane supported NLSV at *two* locations, one where the heated FM is overlaid by the NM channel and the second where the voltage lead (here formed by the same Al layer) connects. These locations are indicated in Fig. 11b), and both have significant in-plane ∇T. Taking both into account allows for accurate determination of the ANE coefficient, $S_{N}$, which is plotted vs. T for two membrane-supported NLSVs using permalloy with different L [156] and compared to the same value determined from a substrate-supported NLSV in the first work that clarified ANE contributions to these devices [139]. The divergence in $S_{N}$ at lower T for the two NLSVs shown in Figure 11 is potentially due to the slightly different geometry used in the detector FM for the two separations, but further work is needed to assess this effect. All values shown here agree well near room temperature, though the broader literature on ANE in permalloy and other 3d ferromagnets shows a somewhat wider range of values [151,154,158,159]. For permalloy specifically these range from values of $S_{N}$ near 0.04μV/K observed by Chuang, *et al*. using out-of-plane thermal gradients induced by heated copper blocks (inspired by the techniques often used to measure the longitudinal SSE) [151], to values near 0.6 μV/K observed by Yamazaki, *et al*. using micropatterned Hall bars on a solid substrate with varying thickness [154], to values nearer 1.6μV/K observed in micro- or nanofabricated Py wires by von Bieren, *et al*. [158]. Where also reported, these studies typically show some variability in the measured Seebeck coefficient and electrical resistivity as well. It is not yet clear if the variability in $S_{N}$ is due to measurement challenges, to extrinsic effects in the material, or to size effects. This remains a key open question in the field.

The ability to perform 2d finite element modeling of suspended Si-N structures to determine the in-plane thermal gradients in magnetic films has also played an important role in measurements of the planar Nernst effect (PNE) [159–165]. This thermal analog to the planar Hall effect (PHE), like the PHE, is driven by the physics governing the scattering of electrons in ferromagnetic metals that also underlies the anisotropic magnetoresistance (AMR). This arises from spin-orbit scattering and results in an electrical resistivity that depends on the angle θ between the local magnetization of a domain and the direction of the charge current passing through it. This takes the form [166]:

(6) $$\rho (\theta )=\rho_{⊥}+\Delta \rho \cos^{2}(\theta ),$$

where Δρ is the AMR ratio defined by:

(7) $$\frac{\Delta \rho }{\rho_{\mathrm{av}}}=\frac{\rho_{∥}-\rho_{⊥}}{\frac{1}{3}\rho_{∥}+\frac{2}{3}\rho_{⊥}},$$

where $\rho_{∥}$ and $\rho_{⊥}$ are the resistivity measured with magnetization parallel and perpendicular to I, respectively. This ratio is commonly several percents in transition metal FM films [166].

The dependence of ρ, measured in the traditional 4-wire configuration with longitudinal voltage connections, arises from variation in scattering from the orbital charge distribution, which remains perpendicular to the magnetization as the domain rotates (an intuitive picture shown in more detail elsewhere [69]). The same scattering drives a transverse flow of charge, which leads to the PHE, which can be explicitly related also to $\rho_{⊥}$ and $\rho_{∥}$:

(8) $$\rho_{\mathrm{PHE}}(\theta )=\frac{1}{2}[\rho_{∥}-\rho_{⊥}]\sin2\theta .$$

If we instead drive the system with an in-plane thermal gradient, the same scattering results in a magnetic field-dependent thermopower (or thermoelectric power), the MTEP, which results in different Seebeck coefficients when the magnetization of a domain is oriented differently with respect to the thermal gradient, giving different values of $S_{⊥}$ and $S_{∥}$. Again there is a transverse flow of charge, leading to the planar Nernst effect (PNE), again related to these thermopowers:

(9) $$S_{\mathrm{PNE}}(\theta )=\frac{1}{2}[S_{∥}-S_{⊥}]\sin2\theta .$$

The transverse electric field generated is then $E_{y,\mathrm{PNE}}=S_{\mathrm{PNE}}(\theta )\mathrm{\partial T}/\mathrm{\partial x}$ and the transverse PNE voltage is

(10) $$V_{T,\mathrm{PNE}}=S_{\mathrm{PNE}(\theta )}\frac{\partial T}{\mathrm{\partial x}}w,$$

where w is the width of the sample in the transverse direction and the thermal gradient (which is one-dimensional to good assumption due to the Si-N membrane geometry) is ∂T/∂x. The expected size of the PNE voltage can therefore be determined from longitudinal Seebeck coefficient measurements in the two fully in-plane magnetized directions. This has been experimentally demonstrated [116,167,168].

**Figure 12.**
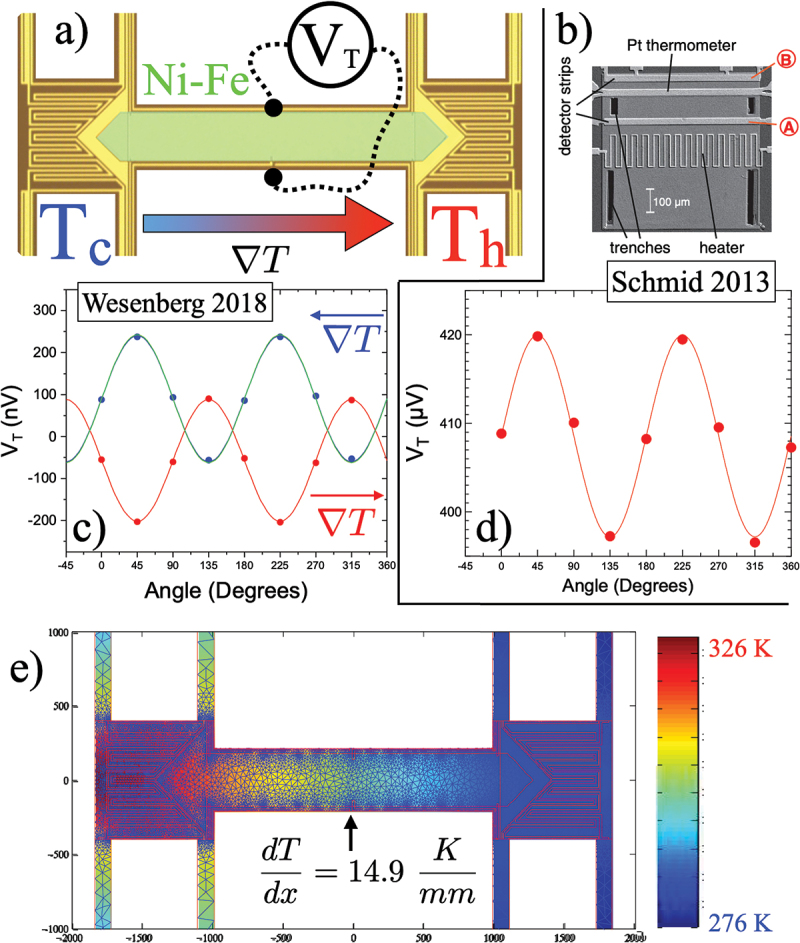
(a) annotated optical micrograph of a Si-N thermal platform optimized for transverse voltage measurements. The location of one set of transverse voltage taps is shown schematically, and the location of a permalloy (Ni-Fe) sample is shown in false color (green) [116]. (b) Scanning electron micrograph, modified from [164] with permission of the authors, copyright 2013 by the American Physical Society, showing a patterned Si-N platform used for PNE measurements of permalloy. c,(d) $V_{T}$ vs. angle θ for the two different PNE measurements shown above [116,164], both showing the sin(2θ) dependence expected for the PNE. In (c) the green line is the predicted $V_{T}$ from measured magnetothermopower and thermal gradient. (e) Calculated thermal profile for the Si-N membrane platform shown in (a), which allows for the determination of the thermal gradient at the location of the transverse voltage taps, as indicated [116].

Two examples of PNE measurements using Si-N membrane thermal platforms are shown in Figure 12, where two groups used somewhat different geometries to examine the PNE in permalloy thin films, as shown in an annotated optical micrograph in panel a) [116], and in a scanning electron micrograph in panel b) [164]. The resulting transverse voltage $V_{T}$ vs. angle θ for the measurements are shown in Figure 12(c, d), respectively. Both show the expected sin2θ dependence of Eq. 9. Wesenberg *et al*. also compared the transverse voltage to the measured MTEP in the same film, which results in the predicted data shown by the green solid line, which is an excellent match to the measured transverse voltage, as also seen in earlier work [163,167]. Figure 12e) shows 2d finite element modeling used to quantify the in-plane thermal gradient in the Si-N/Py beam, which enables excellent agreement between predicted and measured PNE [116].

## 6. Thermal spin injection

Thermal spin injection, as currently understood, requires a fairly sharp thermal gradient, and has only been clearly established experimentally using out-of-plane gradients (or in devices where an out-of-plane gradient is certainly present). These include the important and quite widely studied longitudinal spin Seebeck effect (LSSE) [169–171] and the spin-dependent Seebeck effect (SDSE) [140–149]. The LSSE arises when an out-of-plane thermal gradient on a magnetic insulator (most frequently yttrium iron garnet, YIG) drives an incoherent flow of magnons and its associated spin current that impinges on a metal film with spin-charge conversion, such that the injected spin current drives a transverse charge voltage that is readily measured. The SDSE, which is more challenging to measure and examined somewhat less frequently, occurs when a thermal gradient applied within a spin diffusion length of a metallic ferromagnet/non-magnetic metal interface injects a non-equilibrium spin current into the normal metal. This has most frequently been observed in specially designed metallic non-local spin valves (NLSV) [140–149]. Attempts to observe thermal spin injection using in-plane thermal gradients have been dominated by other thermomagnetic or thermoelectric effects [158,159,163,164,172] though some in the field still debate these issues.

I also note that the expected reciprocal effects for both types of thermal spin injection have now been well documented in experiments. The reciprocal effect of the LSSE is the spin Peltier effect (SPE), where a magnonic spin current injected through the interface between a spin-charge converting metal and a ferromagnetic insulator develops a thermal gradient [173–177]. The reciprocal effect of the SDSE is the spin-dependent Peltier effect, SDPE, where a purely electronic spin current transmitted across an interface between a metallic ferromagnet and a nonmagnetic metal generates a thermal gradient [178,179]. To my knowledge, none of these challenging experiments, which require extremely sensitive thermometry, has taken advantage of Si-N membrane isolation platforms due most likely to materials incompatibility. If materials of interest for SPE and SDPE measurements could be grown on Si-N membranes, these effects could perhaps be more widely explored.

## 7. Specific heat capacity

Heat capacity and differential scanning calorimetry measurements, which as stated earlier were among the first motivations for the design and fabrication of micromachined thermal measurement devices using Si-N membranes, have made important contributions to the fundamental physics and materials properties of many magnetic thin-film systems. These techniques are naturally suited to investigate and identify the temperature of phase transitions, especially in antiferromagnetic or compensated systems where small or vanishing magnetization makes measurements with magnetometry difficult. The thermodynamic signature of magnetic ordering persists even with zero magnetization. The small sample mass of a thin film (now reaching into the range of nanograms or smaller) or highly phase-pure single crystal often also makes neutron scattering, another common method of observing ordering when magnetization vanishes, inaccessible. Examples where Si-N membrane heat capacity measurements have filled this need include studies of NiO/CoO superlattices [180,181], iron-rhodium thin films [182] including examination of epitaxial films [35], amorphous rare-earth-transition metal alloy films [183], antiferromagnetic chromium thin films [184], ultrathin Ni films [185], and a range of complex oxide crystals [186–188]. Si-N thin-film calorimeters have also allowed the investigation of the fundamental nature of electronic and phononic contributions to heat capacity in systems such as GMR-related Fe/Cr multilayers [189], and evolution of magnetic entropy in amorphous silicon-based spin glasses that are impossible to produce in bulk form [190,191]. As new magnetic materials and phases continue to be discovered and prove interesting for spintronic memory and other information technology applications where tiny structures are essential, these heat capacity techniques are well positioned to play increasingly important roles.

## 8. Future outlook

The links between spin, heat, and charge that have fueled the robust interest in spin caloritronics [192–195] continue to be pushed into new frontiers of novel materials, new phenomena, and exciting potential applications. These areas have opened fresh opportunities for the Si-N membrane enabled techniques to contribute. A few particularly exciting new potential areas are ANE studies of more exotic magnetic systems that have proven very exciting in bulk-like measurements of this transverse thermopower, which include Weyl semimetals [196–198], non-collinear antiferromagnets [5,6], and most recently, altermagnets [8,199]. Use of Si-N membranes could dramatically improve the accuracy of ANE measurements, though there is a significant challenge to be met regarding the implementation of Si-N supported platforms that allow for the growth of epitaxial or otherwise highly ordered samples. Techniques to achieve this have been demonstrated for Si-N nanocalorimeters optimized for heat capacity measurements [35,36], but such approaches remain underexploited for k, $S_{\mathrm{abs}}$, and Nernst measurements. Peltier measurements are another area where additional investigation could help elucidate new physics, since many of the more complicated magnetic states listed above involve broken time-reversal symmetries that could prove interesting to investigate through the lens of Onsager reciprocity. Finally, there are many open questions regarding the role of spin dynamics in heat transport in low damping ferromagnets and antiferromagnets of various types and concerning how grain boundaries affect the electron, phonon, and magnon dynamics that govern heat flow in nanoscale magnetic systems. The coming years should remain a fruitful, if challenging, time for this area of magnetism and magnetic materials.
